# Development, Characterization, Stability and Bioaccessibility Improvement of 7,8-Dihydroxyflavone Loaded Zein/Sophorolipid/Polysaccharide Ternary Nanoparticles: Comparison of Sodium Alginate and Sodium Carboxymethyl Cellulose

**DOI:** 10.3390/foods10112629

**Published:** 2021-10-29

**Authors:** Yufeng Chen, Jingchong Peng, Yueqi Wang, Daniel Wadhawan, Lijun Wu, Xiaojing Gao, Yi Sun, Guobin Xia

**Affiliations:** 1 College of Food Science and Technology, Zhejiang University of Technology, Hangzhou 310014, China; wuyycyf@163.com (Y.C.); pjchs6g@163.com (J.P.); wulj416@163.com (L.W.); gaoxiaojing66@163.com (X.G.); sunyi971219@163.com (Y.S.); 2 Key Lab of Aquatic Product Processing, Ministry of Agriculture and Rural Affairs of the People’s Republic of China, South China Sea Fisheries Research Institute, Chinese Academy of Fishery Sciences, Guangzhou 510300, China; 3 Department of Natural Science and Mathematics, University of Houston, Houston, TX 77002, USA; dkwadhawan2@uh.edu; 4 Department of Pediatrics Section of Neonatology, Texas Children’s Hospital, Houston, TX 77030, USA

**Keywords:** Zein, 7,8-dihydroxyflavone, sophorolipid, polysaccharide, ternary nanoparticles

## Abstract

In this study, two polysaccharides [sodium alginate (ALG) and sodium carboxymethyl cellulose (CMC)] were selected to establish zein/sophorolipid/ALG (ALG/S/Z) and zein/sophorolipid/ALG (CMC/S/Z) nanoparticles to encapsulate 7,8-dihydroxyflavone (7,8-DHF), respectively. The results showed that polysaccharide types significantly affected performance of ternary nanoparticles, including CMC/S/Z possessed lower polydispersity index, particle size and turbidity, but higher zeta potential, encapsulation efficiency and loading capacity compared to ALG/S/Z. Compared to zein/sophorolipid nanoparticles (S/Z), both ALG/S/Z and CMC/S/Z had better stability against low pH (pH 3~4) and high ionic strengths (150~200 mM NaCl). Hydrophobic effects, electrostatic interactions and hydrogen bonding were confirmed in ternary nanoparticles fabrication via Fourier-transform infrared spectroscopy. Circular dichroism revealed that CMC and ALG had no evident impact on secondary structure of zein in S/Z, but changed surface morphology of S/Z as observed by scanning electron microscope. Encapsulated 7,8-DHF exhibited an amorphous state in ternary nanoparticles as detected by X-ray diffraction and differential scanning calorimetry. Furthermore, compared to S/Z, ALG/S/Z, and CMC/S/Z remarkably improved the storage stability and bioaccessibility of 7,8-DHF. CMC/S/Z possessed a greater storage stability for 7,8-DHF, however, ALG/S/Z exhibited a better in vitro bioaccessibility of 7,8-DHF. This research provides a theoretical reference for zein-based delivery system application.

## 1. Introduction

7,8-dihydroxyflavone (7,8-DHF) (Figure S1), a naturally occurring infrequent flavone monomeric compound from *Tridax procumbens* and *Godmania aesculifolia*,, was authenticated as a high-affinity tropomyosin receptor kinase B (TrkB) agonist [1,2]. It could mimic the physiological roles of brain-derived neurotrophic factor (BDNF) and its associated downstream signaling pathway [3]. Current literature confirms that 7,8-DHF’s role in attenuating many BDNF- relevant human illnesses, including Parkinson’s disease, Alzheimer’s disease, obesity, and depression [4,5,6,7,8]. However, glucuronidation, sulfation, and methylation of 7,8-DHF in the intestinal tract or liver caused its extremely low oral bioavailability [9]. In our previous study, we testified that 7,8-DHF’s permeability coefficient was lower than 3 × 10^−6^ cm/s and had an active efflux mediated by multidrug resistance-related proteins (MRPs, especially MRP 2 outflow) and P-glycoprotein (P-gp) [10]. Thus, the low chemical instability, poor water solubility, and intestinal efflux of 7,8-DHF limit its application as a nutraceutical ingredient in functional food and beverage products. 

To resolve the previously mentioned limitations and challenges, constructing an effective delivery vehicle using food-grade polymer is a novel strategy for boosting the utilization of water-insoluble functional components, such as liposomes, microemulsions, nano-emulsions, nanosuspensions, and nanoparticles [11,12,13,14]. Among these carriers, protein-based polymers, such as ovalbumin [15], casein [16], gliadin [17], and zein [18], have been utilized to create colloidal delivery systems for phenolic compounds to improve their physicochemical stability, water insolubility, and bioaccessibility. Specifically, these polymers possess several benefits such as high levels of biocompatibility, biodegradability, and label-friendliness.

As a primary storage protein found in corn, zein occupies for approximately 45~50% of total protein content, and has a relatively high percentage of hydrophobic groups. Zein’s high level of hydrophobic groups means that it is soluble in 60~95% ethanol-water solutions, but insoluble in water [19]. The varying solubility of zein indicates that zein colloidal delivery systems can be established by antisolvent precipitation (ASP) [20]. Nevertheless, when exposed to a certain temperature, ionic strength, and pH range, zein nanoparticles are highly susceptible to aggregation because of the strong hydrophobic attractions among them. Previous studies have shown that food-grade biopolymers and surfactants can improve the aggregation stability of zein nanoparticles by establishing a protective layer around them. Among these applications, zein-based ternary nanoparticles exhibited more advantages compared to binary nanoparticles. Dai et al. and Wei et al. report that zein/polysaccharide/surfactant ternary nanoparticles functioned as delivery systems for coenzyme Q_10_ and curcumin, enhancing their structural stability and in vitro bioaccessibility [21,22]. Zhang et al. reported a one-step assembly method for building zein/caseinate/alginate nanoparticles, as propolis were successfully encapsulated in these carriers, their bioaccessibility was improved [23]. In addition, zein/caseinate/sodium alginate nanoparticles were also established to improve the controlled release and physicochemical properties of curcumin [24].

In our previous study, we utilized sophorolipid, a surfactant molecule, to function as fabrication stabilizer for zein binary nanoparticles. These nanoparticles displayed stability at varying salt concentrations (0~100 mM NaCl) and a wide pH range (5~9) [25]. However, at low pH conditions, their stability was low. Therefore, we aim to use biomacromolecules like polysaccharide in this study to construct zein ternary nanoparticles and solve the aforementioned defects. Sodium alginate (ALG) is a natural anionic polysaccharide with a structure of (1 → 4)-b-d-mannan and (1 → 4)-a-l-guluronopyranoys homopolymer sequence isolated from *Phaeophyceae*. ALG can be used as a carrier for biological food components [26]. In addition, sodium carboxymethyl cellulose (CMC) is also a suitable anionic polymer for the delivery of bioactive ingredients [27]. Due to their widespread availability and functional properties, CMC and ALG are favorable candidates for the protection of coated zein/sophorolipid binary nanoparticles when encapsulating 7,8-DHF.

The first objective of our research was to explore the effect of polysaccharides type (CMC and ALG) on the formation and performance of zein ternary nanoparticles. Subsequently, to improve in vitro bioaccessibility and storage stability of 7,8-DHF, zein ternary nanoparticles encapsulation were manufactured. Moreover, the microstructure and chemical structure of these ternary nanoparticles were analyzed by a series of characterization techniques. For precision diet intervention or functional food field, this research provides valuable insights to develop more effective delivery systems for 7,8-DHF, which will allow more 7,8-DHF to be absorbed by the human intestines, exerting greater biological effects for human heath. For functional beverage industry, stable zein-surfactant-polysaccharide delivery systems allow hydrophobic active components, such as 7,8-DHF, to be better dispersed in the water-phase beverage system to develop more functional beverage products.

## 2. Materials and Methods

### 2.1. Materials and Chemicals

Pancreatin (4 × USP specification) and zein (≥95%) and were bought from Sigma-Aldrich (Missouri, USA). 7,8-dihydroxyflavone (≥98%) was purchased from TCI Co., Ltd. (Tokyo, Japan). Sophorolipid was purchased from the Boliante Chemical Company (Xian, China). Sodium alginate (>90%) was obtained from Macklin (Shanghai, China). Sodium carboxymethyl cellulose was purchased from Aladdin (Shanghai, China). Bile salts and pepsin (activity 3000~3500 U mg^−1^) were obtained from Sangon Biotech Co., Ltd. (Shanghai, China). Other utilized reagents and chemicals were of analytical grade.

### 2.2. Zein/Sophorolipid/Polysaccharide Ternary Nanoparticles Preparation

Zein/sophorolipid/polysaccharide ternary nanoparticles and zein/sophorolipid binary nanoparticles (S/Z) were fabricated based on our previous study [25]. In detail, zein and sophorolipid (mass ratio 1:1, *w*/*w*) were both dissolved in 80% ethanol/water solution to prepare the stock solution (1% sophorolipid +1% zein, *w*/*v*). For preparation of zein/sophorolipid/polysaccharide ternary nanoparticles, the stock solution was quickly added into the polysaccharide (ALG or CMC) aqueous solution (antisolvent) in a volume ratio of 1:3 (*v*/*v*) under continuous stirring at 800 rpm for 30 min. Subsequently, ethanol was removed by a rotary evaporator at appropriate temperature. The mass ratio (*w*/*w*) of zein to polysaccharide was set to 30:1, 20:1, 10:1, 5:1, 3:1, 2:1, and 1:1, respectively. The prepared CMC-based ternary nanoparticles were expressed as CMC/S/Z 30:1, CMC/S/Z 20:1, CMC/S/Z 10:1, CMC/S/Z 5:1, CMC/S/Z 3:1, CMC/S/Z 2:1, and CMC/S/Z 1:1, respectively. And the ALG-based ternary nanoparticles were denominated as ALG/S/Z 30:1, ALG/S/Z 20:1, ALG/S/Z 10:1, ALG/S/Z 5:1, ALG/S/Z 3:1, ALG/S/Z 2:1, ALG/S/Z 1:1, respectively. In addition, the S/Z was also fabricated by ASP methods; the difference was that antisolvent was deionized water without ALG or CMC. The final concentration of zein in every nanoparticle was 2.5 mg/mL. Then, the pH value of each nanoparticle dispersion was adjusted to 4.0 for dispersions stability investigation. The CMC/S/Z 5:1 and ALG/S/Z 5:1 were used for the following structure characterization. The freshly prepared samples were stored at 4 °C, and a portion of each sample was freeze-dried into lyophilized powder for the following testing and analysis.

### 2.3. Polydispersity Index (PDI), Particle Size, Zeta Potential and Turbidity

PDI, particle size, and zeta potential of fresh dispersions were characterized using a dynamic light scattering (DLS) instrument (Nano-ZS90 analyzer, Malvern, UK) at 25 °C. PDI and particle size was detected via light intensity at a fixed scattering angle of 90°, and the refractive index of water was set at 1.45. The zeta potential was calculated by Smoluchowski model. The turbidity of complex particles was tested at 600 nm using an ultraviolet-visible spectrophotometer at 25 °C.

### 2.4. Physical Stability of Ternary Nanoparticles

#### 2.4.1. pH Influence

The influence of pH on physical stability of each nanoparticle dispersion was evaluated within a pH range of 3 to 9 adjusting by either 2 M HCl or 2 M NaOH.

#### 2.4.2. Ionic Strength Influence

Each nanoparticle dispersion was blended with NaCl to obtain samples with 0, 25, 50, 100, 150 and 200 mM NaCl concentrations and stored for 24 h for observing physical stability.

Size changes within nanoparticle dispersions were recorded using DLS at 25 °C.

### 2.5. Encapsulation of 7,8-DHF into Ternary Nanoparticles

7,8-DHF encapsulation was conducted according to the methods described in Section 2.2. 7,8-DHF, sophorolipid, and zein were dissolved at a mass ratio of 1:10:10 and 1:5:5 in 80% ethanol/water solution, respectively. The mass ratio of zein to polysaccharide was 5:1 in the final reaction system. 7,8-DHF encapsulation in binary and ternary nanoparticles were denoted as DHF-S/Z, DHF-CMC/S/Z, and DHF-ALG/S/Z. Loaded complex particles were reserved at 4 °C, with other samples freeze-dried for 48 h to conduct further analysis.

### 2.6. Encapsulation Efficiency (EE) and Loading Capacity (LC)

The EE and LC of encapsulated 7,8-DHF were assessed by UPLC based on our previously described method [25]. Specifically, the nanoparticles were centrifuged at 10,000× *g* for 10 min at 4 °C using a centrifugal machine (3K15, Sigma, Osterode, Germany). The supernatant (containing loaded 7,8-DHF) was removed and diluted 5-fold with methanol. And an equal volume of the initial suspension was diluted in 5-fold methanol to obtain initial 7,8-DHF. Then, EE and LC were calculated according to the following equation:

EE (%) = loaded 7,8-DHF/initial 7,8-DHF × 100(1)

LC (%) = loaded 7,8-DHF/weight of vehicle × 100(2)

### 2.7. 7,8-DHF Loaded Ternary Nanoparticles Characterization

#### 2.7.1. Fourier-Transform Infrared (FTIR) Spectroscopy

7,8-DHF and lyophilized sample under analysis were prepared by adding 99% KBr disc and scanned on an FTIR spectrometer (Avatar 370, Nicolet, Madison, WI, USA). The spectral scanning range was 500~4000 cm^−1^ at a resolution of 4 cm^−1^. The analytical results were carried out by OMNIC software version 8.0.

#### 2.7.2. Circular Dichroism (CD) Spectrum

Secondary structural characteristics of nanoparticle dispersions under analysis were measured using a CD spectrometer (J-1500, JASCO, Tokyo, Japan). The concentration of complex dispersions was 0.2 mg/mL corresponding to zein. The buffer in CD experiments was deionized water (pH = 7). The secondary structure scanning region was 190~260 nm with a 0.1 cm path length. The bandwidth was 1.0 nm and scanning speed was 50 nm/min. The data were evaluated by Spectra Manager™ II Software equipped with CD spectrometer.

#### 2.7.3. Differential Scanning Calorimetry (DSC)

Thermal behavior of 7,8-DHF and freeze-dried samples were studied via DSC (Mettler Toledo, Zurich, Switzerland). 2~10 mg of samples were accurately weighed and hermetically sealed in aluminum pans. An empty crucible under the same condition was used as a reference. Scanning calorimetry was performed from a range of 25 to 200 °C in N2 atmosphere at a heating rate of 10 °C /min under 30 mL/min flow.

#### 2.7.4. X-ray Diffraction (XRD)

The crystalline characteristic of lyophilized nanoparticles and selected 7,8-DHF were recorded on an X-ray diffractometer (Bruker D8, Karlsruhe, Germany). This diffractometer was carried at 40 kV accelerating voltage and 40 mA tube current to produce copper Kα radiation. Soller slit and divergence slit were set at 2.5° and 0.5°, respectively, the 2θ angle was ranged from 5° to 90°.

#### 2.7.5. Transmission Electron Microscopy (TEM)

10-fold diluted fresh nanoparticle dispersions were deposited on a copper grid with formvar-carbon coating. Then, the samples were air-dried for 5 min and stained with 2% uranyl acetate. TEM (JEM-1200 EX, Tokyo, Japan) was performed for microscopic observation at 120 kV accelerating voltage.

#### 2.7.6. Field Emission Scanning Electron Microscope (FE-SEM) 

The surface morphology of polysaccharide samples and freeze-dried nanoparticles was captured by FE-SEM (GeminiSEM 300, ZEISS, Germany). Before analysis, 3–6 nm of a thick gold layer was covered on the sample surfaces. The electron microscope acceleration voltage was 15.0 kV.

### 2.8. Storage Stability of 7,8-DHF

7,8-DHF, DHF-S/Z, DHF-ALG/S/Z, and DHF-CMC/S/Z were performed at 5 °C for 72 h under dark and 25 °C for 15 days under light, respectively. At an appropriate point in time, the samples were acquired with 7,8-DHF presence being measured by UPLC. The storage stability was calculated based on the following equation:

Retention rate (%) = retained 7,8-DHF concentration/initial 7,8-DHF concentration × 100(3)

### 2.9. In Vitro Simulated Gastrointestinal Digestion

Based on Yuan et al.’s study via some amendments [28], briefly, 10 mL of simulated gastric fluid (SGF, 3.2 mg/mL pepsin and 2 mg/mL NaCl, pH = 2.5) and 10 mL of 7,8-DHF, DHF-S/Z, DHF-ALG/S/Z, and DHF-CMC/S/Z were mixed for incubating in a 37 °C water bath shaker for 60 min at 100 rpm. After SGF digestion, 10 mL of above-mentioned simulated gastric digestive fluids were rapidly adjusted to pH 7.4 using 2 M NaOH. Whereafter, 10 mL of simulated intestinal fluid (SIF, 4 mg/mL pancreatin, 5 mg/mL bile salts, 6.8 mg/mL K2HPO4 and 8.8 mg/mL NaCl, pH = 7.4) was added into above-mentioned simulated gastric digestive fluids and incubated for 120 min at same temperature and revolving speed. Finally, the final digestive solution was centrifuged by 20,000× *g* centrifugal force for 1 h, and the supernatant (the mixed micelle phase containing 7,8-DHF) was collected. The bioaccessibility (%) was calculated based on the following equation:

Bioaccessibility (%) = 7,8-DHF concentration in the micelles phases/7,8-DHF concentration in the formulation × 100(4)

### 2.10. Statistical Analysis

Mean ± SD was presented via at least three times for all data. One-way ANOVA followed by Tukey’s honestly significant difference *post hoc* tests were utilized to assess potential significant differences among groups. A *p* < 0.05 indicated significant differences between groups. Data analysis was carried out by Origin 2021 (Origin Lab Co., Northampton, MA, USA) and GraphPad Prism version 8.0 (GraphPad Sofware, San Diego, CA, USA).

## 3. Results and Discussion

### 3.1. Effect of Polysaccharides on Ternary Nanoparticles Fabrication

**Figure 1:**
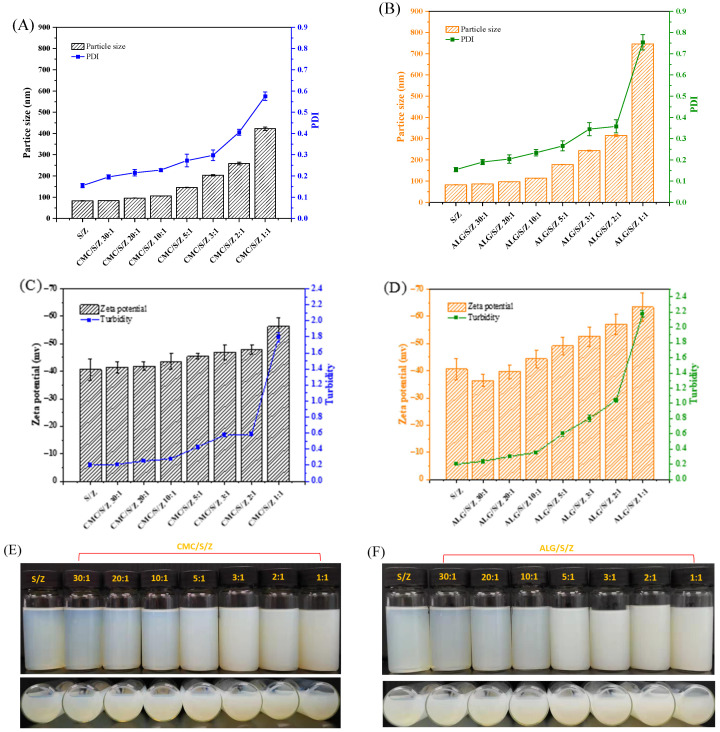
Particle size, PDI, zeta potential and turbidity of CMC/S/Z and ALG/S/Z with different zein to polysaccharide mass ratios. Particle size and PDI (**A**,**B**). Zeta-potential and turbidity (**C**,**D**). The photograph of each group appearance (**E**,**F**).

The effects of varying concentrations and types of polysaccharide on particle size of S/Z were illustrated in Figure 1. S/Z was approximate 82.81 nm particle size. The particle size of the ternary nanoparticles was differentially altered from that of S/Z after introduction with CMC and ALG into the binary system. After supplementation with CMC, the particle size of CMC/S/Z stepwise grew as the concentration of CMC increased (Figure 1A). Moreover, the incorporation of ALG resulted in a significant increase in particle size (*p* < 0.05). And in comparison to CMC/S/Z, ALG/S/Z showed a greater particle size at the identical polysaccharide level (Figure 1B). The above phenomenon might be attributed to the distinctive molecular structure of two studied polysaccharides and interaction force among molecules. The changes in particle size of zein/sophorolipid/polysaccharide ternary nanoparticles are possibly a result of sufficient anionic CMC and ALG being absorbed on the surface of S/Z through the electrostatic attractions. The changes in PDI value of CMC/S/Z and ALG/S/Z was analogous to that of particle size. As the concentration of CMC and ALG increased, the PDI value of CMC/S/Z and ALG/S/Z gradually increased as well. However, ALG/S/Z showed a higher PDI value at the same polysaccharide level.

The zeta-potential of S/Z was −40.63 mV. When CMC and S/Z were combined, their charge values became more negative as the fraction of present CMC increased (Figure 1C). On the other hand, the negative charges of ALG/S/Z initially declined and grew whereafter when the level of ALG was increased (Figure 1D). When the mass ratio of polysaccharide to zein was above 1:10, the negative charges of ALG/S/Z were higher than that of CMC/S/Z. This difference is potentially caused by the different molecular structures of the two polysaccharides. The zeta-potential of zein/sophorolipid/polysaccharide ternary nanoparticles had been dominated by negatively charged CMC and ALG. It is reported that electrostatic attraction played a critical role in the formation of protein-surfactant-polysaccharide complexes, for instance, zein-rhamnolipid-propylene glycol alginate [29], and zein-lecithin-propylene glycol alginate [30]. Meanwhile, the uniform trend of turbidity in ternary nanoparticles was consistent with that of PDI value and particle size, as the increasing trend of turbidity was primarily caused by micro-aggregation of complexes [31].

### 3.2. CMC and ALG Prevents S/Z Ternary Nanoparticles Precipitation at pH = 4

**Figure 2:**
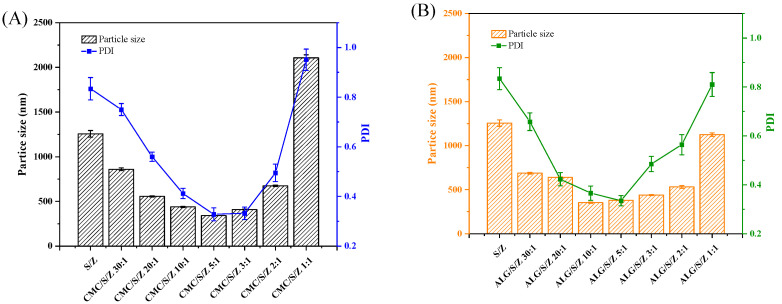

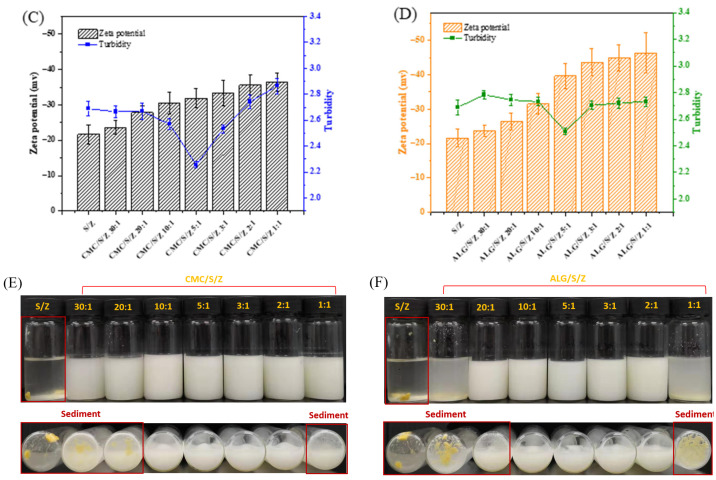
Particle size, PDI, zeta potential and turbidity of CMC/S/Z and ALG/S/Z with different zein to polysaccharide mass ratios at pH = 4. Particle size and PDI (**A**,**B**). Zeta-potential and turbidity (**C**,**D**). The photograph of each group appearance (**E**,**F**).

Our previous work confirms that S/Z exhibits weak stability in low pH conditions. To overcome this deficiency, we explore the influence of polysaccharide type (CMC and ALG) and concentration on S/Z stability at pH = 4. As shown in (Figure 2A), S/Z ternary nanoparticles were extremely unstable and aggregated together as expected. This phenomenon could be elucidated by the lack of appropriate electrostatic repulsion, which is required to conquer attractive interactions (e.g., van der Waals) among nanoparticles. Furthermore, at low pH values, the pKa of hydrophilic sugar residues present on sophorolipid molecules were reported to promote this instability [32]. After CMC was added, the PDI value, particle size, and turbidity of CMC/S/Z initially declined and subsequently jumped until a mass of zein to CMC was 5:1, the CMC/S/Z were possessed the lowest values for particle size, PDF, and turbidity (particle size ≈ 341.50 nm, PDI ≈ 0.335 and turbidity ≈ 2.257) (Figure 2A,C). Moreover, the incorporation of ALG similarly decreased and then increased PDI value, particle size and turbidity of ALG/S/Z. The particle size for ALG/S/Z was smallest at the 10:1 (353.62 nm), while the PDI value (0.335) and turbidity (2.509) were lowest at 5:1 mass ratio of zein to ALG (Figure 2B,D). As seen from photograph (Figure 2E,F), no floccules were observed at the bottom of the container at varying zein to polysaccharide mass ratios (10:1, 5:1, 3:1, and 2:1). A reason for lacking floccules at these mass ratios might be a result of appropriate CMC and ALG participation, which potentially increased the steric or electrostatic repulsions for S/Z against aggregation. Nevertheless, at a zein to polysaccharide mass of 1:1, there were sediments in both CMC/S/Z and ALG/S/Z. The underlying mechanism for these observations is bridging flocculation, i.e., the ability of a single CMC or ALG molecule to adsorb to the surfaces of two or more S/Z. Therefore, CMC or ALG adsorption results in the formation of clusters [33]. Furthermore, with the CMC and ALG concentrations raised, the charge values of CMC/S/Z and ALG/S/Z became increasingly negative. The intensification of these charges revalidates the idea that CMC/S/Z and ALG/S/Z experience sufficient electrostatic repulsion to avoid precipitation at varying mass ratios of 10:1~2:1. Based on these results, we subsequently selected CMC/S/Z and ALG/S/Z at a mass ratio of zein to polysaccharide at 5:1 as the optimum ratio to study in the following research.

### 3.3. Physical-Chemical Stability Study on Ternary Nanoparticles

The optimum delivery system for food nutraceuticals must accommodate flexible pH and ionic environments during beverages processing, storage, and gut passage. Therefore, pH and salt stability testing of delivery systems is necessary to evaluate colloidal particles’ functionality.

#### 3.3.1. Effect of pH

**Figure 3:**
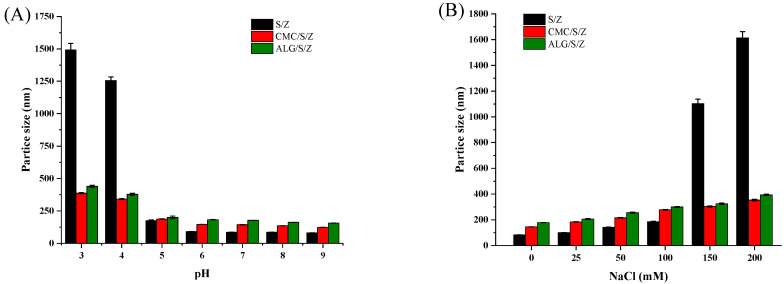
Particle size of colloidal particles at different pH conditions (**A**) and different NaCl concentrations (**B**).

As seen from Figure 3A, S/Z exhibited extensive aggregation at pH 3.0 and 4.0, exhibiting an increased particle size. This increase in size could be due to the fact that weakened electrostatic repulsion was weakened among binary nanoparticles. However, after adding CMC and ALG, compared to S/Z, the stability of both nanoparticles was enhanced noteworthily within pH 3.0 to 4.0. Most notably, from pH 3 to 9, both CMC/S/Z and ALG/S/Z had excellent stability. It is reported that certain zein-surfactant or zein-polysaccharide binary nanoparticles, such as zein-rhamnolipid, zein-alginate, and zein-chondroitin sulfate, experienced a significant size increase at low pH levels [34,35,36]. Unexpectedly, zein-surfactant-polysaccharide ternary nanoparticles exhibited superior pH stability. There are some yellow floccules in the bottom of the container of S/Z at pH 3.0 and 4.0 but no floccules in CMC/S/Z and ALG/S/Z (Figure S4).

#### 3.3.2. Effect of Ionic Strengths

Particle size of S/Z was relatively low by 0 to 100 mM NaCl concentrations (Figure 3B). However, when increased to higher ionic strengths at 150~200 mM NaCl concentrations, precipitates were produced along with increasing particle size. This forming aggregation patterns for nanoparticles at high ionic strengths could be explained by increasing counterions (such as Cl^−^ and Na^+^), depressed electrostatic repulsion via electrostatic screening and nanoparticle charge neutralization [21]. However, in the existence of CMC and ALG, the particle size of CMC/S/Z and ALG/S/Z was increased with the NaCl concentration increased. And adding CMC and ALG reduced the sensitivity of S/Z to aggregation under high ionic strength condition. There are two possible reasons for the improved physical stability of CMC/S/Z and ALG/S/Z. Firstly, CMC and ALG prevented counterion neutralization and strong electrostatic repulsions, which could potentially restrain complex particles from coalescence via enhancing repulsive forces among particles. Secondly, an increase of the steric repulsion among particles could have occurred as polysaccharide molecules were attached to S/Z. Some floccules were also observed at the bottom of the tube for S/Z at 150 and 200 mM NaCl (Figure S5). However, there were no floccules at the bottom of the tube for CMC/S/Z and ALG/S/Z, in which stable colloidal systems were both developed.

### 3.4. Encapsulation of 7,8-DHF

The influence of polysaccharide types (CMC and ALG) and 7,8-DHF concentration on PDI value, particle size, EE and LC value in different delivery systems were summarized in Table 1. When the mass ratio of 7,8-DHF to zein was 1:5, the EE and LC of DHF-S/Z were 82.42 and 7.49%, respectively. However, compared to DHF-S/Z, DHF-CMC/S/Z and DHF-ALG/S/Z exhibited better EE value, especially DHF-CMC/S/Z nanoparticles (88.63%) (*p* < 0.05). These findings suggested that polysaccharide addition improved the embedding ability of S/Z via non-covalent interactions. There are two hydroxyl groups in the 7,8-DHF molecule that can interact with the hydroxyl groups of polysaccharides and the tyrosyl of zein. These interactions can form hydrogen bonds, possibly resulting in an increase of EE. These phenomena indicated that polysaccharides and S/Z showed a synergistic effect on the EE of 7,8-DHF. When mass ratio of 7,8-DHF to zein was 1:10, three colloidal delivery systems possessed high EE for 7,8-DHF (above 98.21%). In contrast to unloaded colloidal delivery systems, the particle size of loaded colloidal delivery systems was growing when increasing the mass ratio of 7,8-DHF to zein. In general, 7,8-DHF, as a hydrophobic compound, is expected to be located within hydrophobic region of zein, ultimately possessing an impact on the size and stability of particles. 7,8-DHF encapsulation might impair interactions among hydrophobic groups of zein and increase particle size. Additionally, the encapsulation of 7,8-DHF caused an increase of PDI value in contrast to unloaded complexes. A uniform result was reported by Sun et al. [37]. The formation of free 7,8-DHF aggregates might help to explain the above observed phenomenon.

### 3.5. Characterization of Loaded Nanoparticles

#### 3.5.1. FTIR

**Figure 4:**
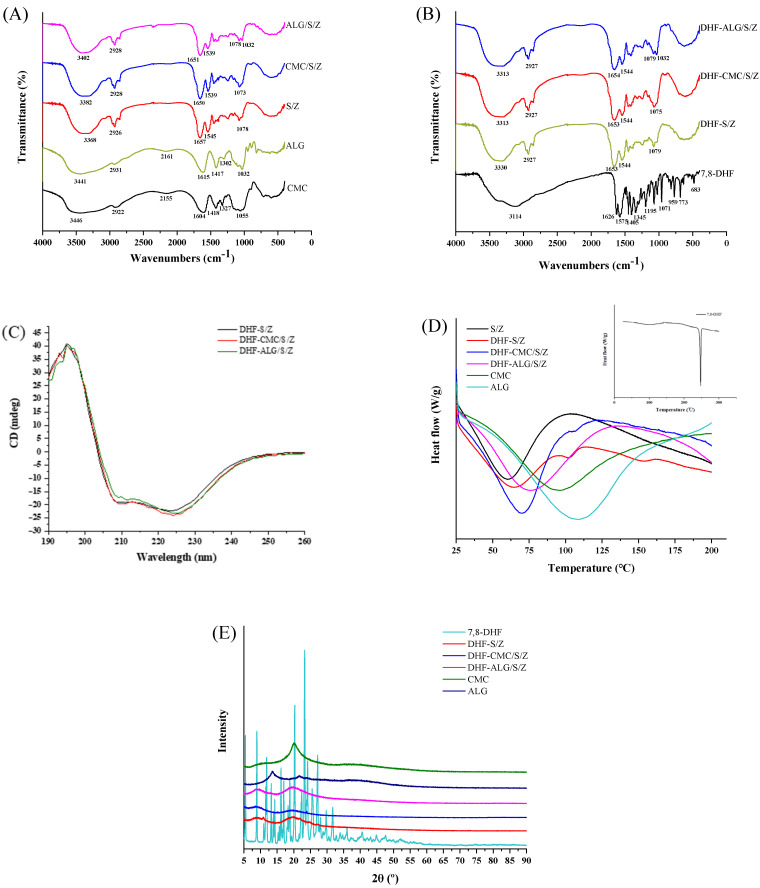
FTIR spectra of CMC, ALG and bare complex nanoparticles (**A**), free 7,8-DHF and 7,8-DHF loaded in each complex nanoparticles (**B**), CD spectra of zein in DHF-Z, DHF-Z/S-CMC, and DHF-ALG/S/Z (**C**), DSC analysis of free 7,8-DHF, CMC, ALG, S/Z, and 7,8-DHF loaded each complex nanoparticles (**D**), XRD spectra of free 7,8-DHF, polysaccharides and loaded each complex nanoparticles (**E**).

FTIR is a versatile tool for monitoring changes within the functional groups of biopolymers and evaluating intermolecular interactions between components and particles. As shown in Figure 4A, the spectrum of CMC and ALG included diversiform representative carbohydrate peaks. The broad 3446 and 3441 cm^−1^ peaks represented hydroxyl groups (O-H) stretching, and the sharp 1604 and 1615 cm^−1^ peaks are associated with carboxyl (-COO-) symmetrical stretching vibration [38]. The featured peaks of CMC and ALG spectra appear at 1418 and 1417 cm^−1^, corresponding to rhamnogalacturonan moiety [39]. Multiple simultaneous vibration peaks in CMC and ALG at 900~1350 cm^−1^ were accredited to characteristic peaks of polysaccharides [24]. In our previous study, we confirmed that there are electrostatic attractions, hydrophobic interactions, and hydrogen bonding in S/Z. The FTIR spectrum of S/Z nanoparticles showed typical characteristic peaks at 3368, 1657, and 1545 cm^−1^, respectively. When CMC and ALG were added, the peaks of O-H stretching vibration (3100~3500 cm^−1^ peaks) shifted from 3368 (S/Z) to 3382 (CMC/S/Z) and 3402 cm^−1^ (ALG/S/Z) [40]. This characteristic peak migration implied that there was a strong hydrogen bond between -OH groups in polysaccharides and amide group of glutamine in zein [41]. According to Liu et al.’s report [42], the 1657 cm^−1^ peak of zein at was the C=O stretching (amide I). 1545 cm^−1^ peak was primarily associated with bending of N-H coupled with the stretching of C-N (amide II). With CMC and ALG incorporation, the amide I and amide II characteristic peaks of ternary nanoparticles were switched to (1650 and 1651 cm^−1^) and (1544 and 1544 cm^−1^), respectively. These results revealed electrostatic attractions were related to the establishment process of CMC/S/Z and ALG/S/Z. Based on these results, we confirmed that ALG/S/Z possessed stronger hydrogen bonding and electrostatic attraction than CMC/S/Z. As seen from Figure 4B, the peaks at 3114, 1626, 1575, 1405, 1195, and 1071 cm^−1^ were the typical peaks of 7,8-DHF, which have been confirmed in our previous study [25,43]. Expectedly, these characteristic peaks of 7,8-DHF were vanished in both binary and ternary nanoparticles, indicating that DHF-S/Z, DHF-CMC/S/Z, and DHF-ALG/S/Z samples successfully encapsulated for 7,8-DHF.

#### 3.5.2. CD Spectrum

In this study, CD spectrum analysis was applied to measure conformational changes (secondary structure) of zein in complexation (260 nm~190 nm). As seen from (Figure 4C), two peaks at 209 and 223 nm with a zero-crossing were around 203 nm (typical secondary structure) in the zein spectrum [44]. As shown in (Table 2), the α-helix, β-sheet and β-turn content of DHF-S/Z changed from 25.3 to 24.7 and 24.5%, 25.8 to 26.1 and 26.6% and 28.3 to 29.1 and 27.9%, respectively after adding CMC and ALG. These results suggested that the addition of CMC and ALG had no apparent impact on zein’s secondary structure in DHF-S/Z. The behavior of CMC and ALG could possibly be the result of sophorolipid adsorption on the zein surface. This adsorption could have possibly resulted in insufficient contact between the polysaccharides and zein, which was difficult to further change the secondary structure of zein. In general, the amide I band shifts in FTIR spectra could also reflect secondary structure changes of the protein, including the band of α-helix, β-sheet, and β-turn [45]. In the FTIR spectra of our study (Figure 4B), the band of amide I did not show significant change among DHF-S/Z, DHF-CMC/S/Z and DHF-ALG/S/Z. This lack of significant change further confirmed that CMC and ALG did not impact the secondary structure of zein.

#### 3.5.3. DSC

Thermal properties of individual components and composite nanoparticles were studied via DSC. As shown in (Figure 4D), the embedded thermograms of 7,8-DHF displayed a narrow and sharp peak at 246.24 °C. This temperature peak was probably caused by the melting of 7,8-DHF crystals [46]. Furthermore, the representative endothermal peak of CMC and ALG was at approximately 96.33 and 113.33°C, respectively. These findings confirmed that ALG had a higher thermostability than CMC because of its specific carbohydrate structure. However, the endothermic peak of S/Z was at approximately 60.33 °C, showing a low thermostability. After 7,8-DHF was encapsulated, no endothermic peaks of 7,8-DHF were found in DHF-S/Z, DHF-CMC/S/Z, and DHF-ALG/S/Z. The lack of endothermic peaks verified that 7,8-DHF was defined as an amorphous form rather than a crystalline form. Similar literature has reported in recent study on curcumin [47] and hyperoside [48]. In addition, the endothermic peak of DHF-S/Z nanoparticles was increased from 60.33 °C to 64.66 °C compared to S/Z. This increase in melting temperatures could be attributed to intermolecular interactions among 7,8-DHF, zein and sophorolipids [29]. Most importantly, after adding CMC and ALG, the endothermic peak of DHF-CMC/S/Z and DHF-ALG/S/Z rose to 70.01 and 76.33 °C in comparison to DHF-S/Z, respectively. The higher endothermic peak of DHF-CMC/S/Z and DHF-ALG/S/Z manifested that they possessed better thermal stability than DHF-S/Z. This new peak might be the result of CMC and ALG interaction enhancements for hydrophobic, electrostatic, or hydrogen bond interactions among different components in nanoparticles, which ultimately leads to a higher endothermic peak temperature.

#### 3.5.4. XRD

X-ray diffraction ranging from 5° to 90° at 2θ values was applied to examine 7,8-DHF’s physical state in different nanoparticles. As presented in (Figure 4E), 7,8-DHF was highly crystalline with multiple sharp diffraction peaks at the range of 5~40°. On the one hand, when the diffraction angles of ALG in the 2θ range were 13.2° and 21.8°, two flat peaks without sharp diffraction maximum appeared in the XRD spectrum. On the other hand, CMC only had a flat peak at a diffraction angle of 20.2°, while no sharp diffraction peaks were emerged. This XRD hump indicated that both pure polysaccharides were totally in an amorphous form [21,32]. However, no obvious sharp diffraction peaks about crystalline form for 7,8-DHF were found in both binary and ternary nanoparticles. Such behavior indicated 7,8-DHF was completely loaded into nanoparticles with an amorphous state. Interestingly, in comparison to DHF-S/Z, the hump for DHF-ALG/S/Z was distinctly increased, while the hump of DHF-CMC/S/Z was only slightly increased as the diffraction angle was at 19.6°. Thus, the polysaccharide types applied to construct nanocomposites can significantly affect XRD patterns. Moreover, no characteristic peaks for these two polysaccharides were observed. Collectively, due to changes in the interactions (hydrophobic, hydrogen bonding, and electrostatic) among zein, polysaccharide, and surfactant molecule, distinguishable behaviors of 7,8-DHF in DHF-S/Z, DHF-CMC/S/Z, and DHF-ALG/S/Z were observed.

### 3.6. Micromorphology

**Figure 5:**
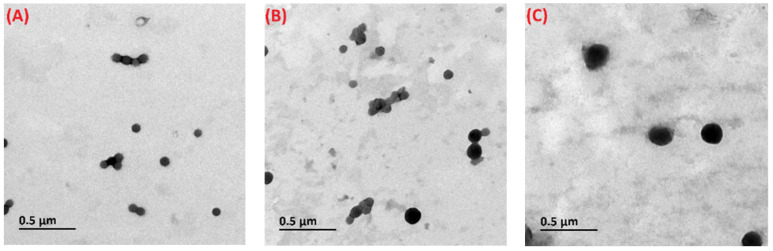
TEM images of DHF-S/Z (**A**), DHF-CMC/S/Z (**B**) and DHF-ALG/S/Z (**C**), 30,000× magnification times.

The microstructural features of loaded composite nanoparticles were analyzed via TEM. As observed in (Figure 5A), the diameter of DHF-S/Z was roughly 100 nm, this finding was in agreement with the results of dynamic light scattering. After adding CMC and ALG, DHF-CMC/S/Z and DHF-ALG/S/Z showed a similar spherical structure that was comparable to the DHF-S/Z. However, the diameter of DHF-CMC/S/Z and DHF-ALG/S/Z was larger than that of DHF-S/Z, exhibiting a particle size of 100~200 nm (Figure 5B,C). These findings demonstrate that CMC and ALG were absorbed on the surface of DHF-S/Z. Additionally, the diameter of DHF-CMC/S/Z was larger than that of DHF-ALG/S/Z, which was consistent with the results of DLS.

**Figure 6:**
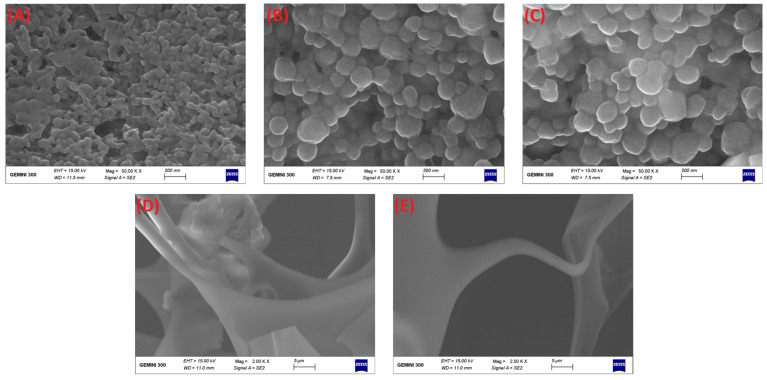
FE-SEM images of DHF-S/Z (**A**), DHF-CMC/S/Z (**B**), DHF-ALG/S/Z (**C**) (50,000× magnification times), individual CMC (**D**) and ALG (**E**) (2000× magnification times).

Furthermore, FE-SEM was applied to further observe the differences in surface microscopic morphology among individual composite nanoparticles (Figure 6). As seen from (Figure 6A), the surface morphology of DHF-S/Z expressed an irregular and rough shape consisting of many interlinked independent complexes. This morphological change was possibly attributed to sophorolipid adsorption on the surface of zein particles. The microphotograph of CMC and ALG depicted a shape similar to that of a silk ribbon (Figure 6D,E). When CMC and ALG were incorporated into DHF-S/Z, the micromorphology of DHF-CMC/S/Z and DHF-ALG/S/Z were spherical with uniform size and smooth surface (Figure 6B,C). The particle sizes of DHF-CMC/S/Z and DHF-ALG/S/Z were larger than that of DHF-S/Z. The polysaccharide coating on the outer layer of DHF-S/Z nanoparticles via electrostatic attraction possibly caused this size difference [49]. The above results were consistent with DLS measurements.

### 3.7. A Graphic Illustration for the Formation and Stability Mechanism of Nanoparticles

**Figure 7:**
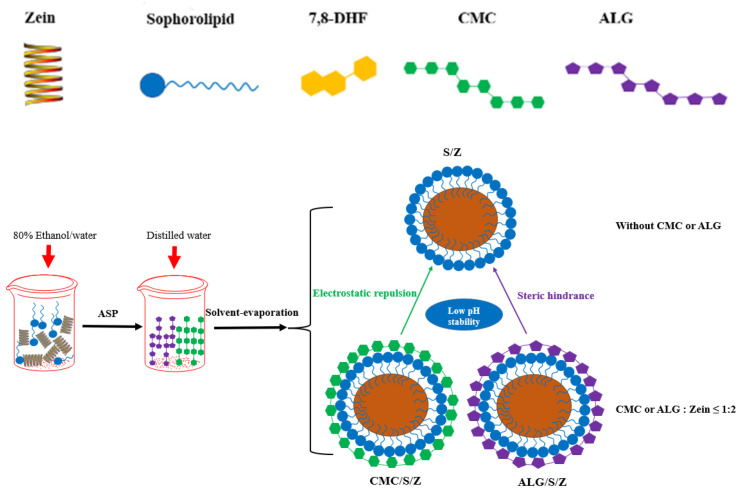

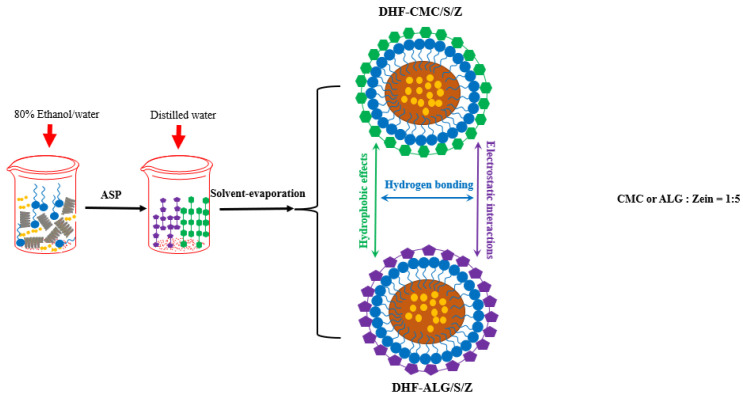
An illustration of the formation and stability mechanism of DHF-CMC/S/Z and DHF-ALG/S/Z.

Diverse technologies and measurements including EE, PDI, particle size, zeta potential, turbidity, CD, TEM, FE-SEM, DSC, XRD, and FTIR were used to make clear the formation and stability mechanism of zein/sophorolipid/polysaccharide ternary delivery system (Figure 7). After zein and sophorolipid were rapidly added into polysaccharide (polysaccharide: zein mass ratio ≤ 1:2), sufficient CMC or ALG acted as a shielding effect (electrostatic repulsion) and steric hindrance stabilizer was coated onto the surface of S/Z particles to prevent their sedimentation at low pH 3~4 range condition, showing a homogeneous PDI, turbidity and size based on DLS and UV. Certain internal drives (electrostatic interaction, hydrogen bonding and hydrophobic effect) participated in the formation of DHF-CMC/S/Z and DHF-ALG/S/Z, and CMC and ALG had no significant impact on secondary structure of zein in S/Z according to CD, FTIR, and FE-SEM. Upon employing EE, XRD, DSC, TEM analysis, 7,8-DHF was shown to be successfully encapsulated in CMC/S/Z and ALG/S/Z with relatively uniform sphericity, displaying a good entrapment efficiency.

### 3.8. Storage Stability of 7,8-DHF

**Figure 8:**
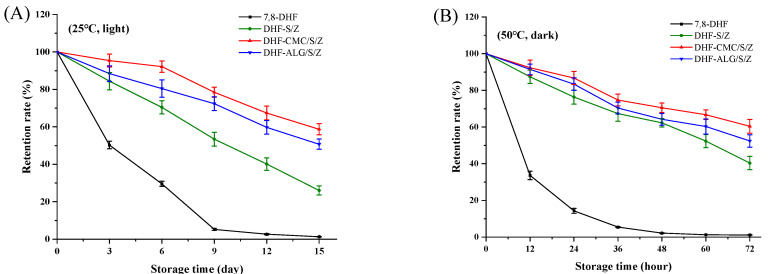
Storage stability of free 7,8-DHF, DHF-S/Z, DHF-CMC/S/Z and DHF-ALG/S/Z at 25 °C under light (**A**), at 50 °C under dark (**B**).

During storage, preventing food neutraceuticals from heat or light exposure is challenging, but critically necessary for mitigating degradation. To meet this application end, short and long term storage were investigated under varying environments for 7,8-DHF, loaded binary and ternary nanoparticles. As shown in (Figure 8A), free 7,8-DHF was mostly degraded at 25 °C with light exposure for 15 days post-storage. Encapsulation of the 7,8-DHF in S/Z nanoparticles strengthened storage stability of 7,8-DHF (26.26 %). The addition of CMC and ALG further enhanced the stability of encapsulated 7,8-DHF, in particular DHF-CMC/S/Z (58.75%). At 50 °C under dark conditions (Figure 8B), a similar effect was observed. The active groups of 7,8-DHF were possibly protected within the hydrophobic lumen of DHF-S/Z, DHF-CMC/S/Z, and DHF-ALG/S/Z nanoparticles as a mechanism [50], besides, due to the different chemical structures of CMC and ALG, DHF-CMC/S/Z have higher EE, which caused more 7,8-DHF to be protected in the hydrophobic part. These results are in agreement with previous studies that introduced curcumin being embedded in zein and quaternized chitosan complexes [51], along with work showing that quercetagetin was loaded using the zein-hyaluronic acid binary complexes [52].

### 3.9. In Vitro Simulated Gastrointestinal Digestion

**Figure 9:**
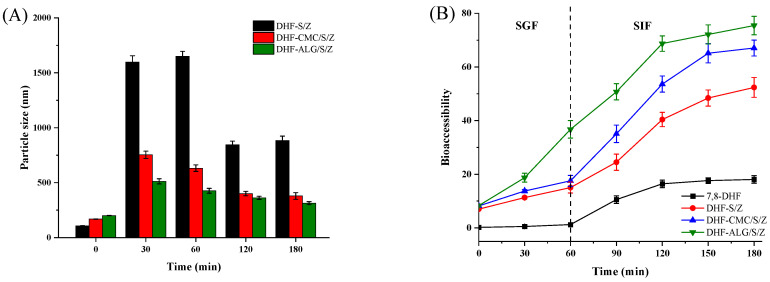

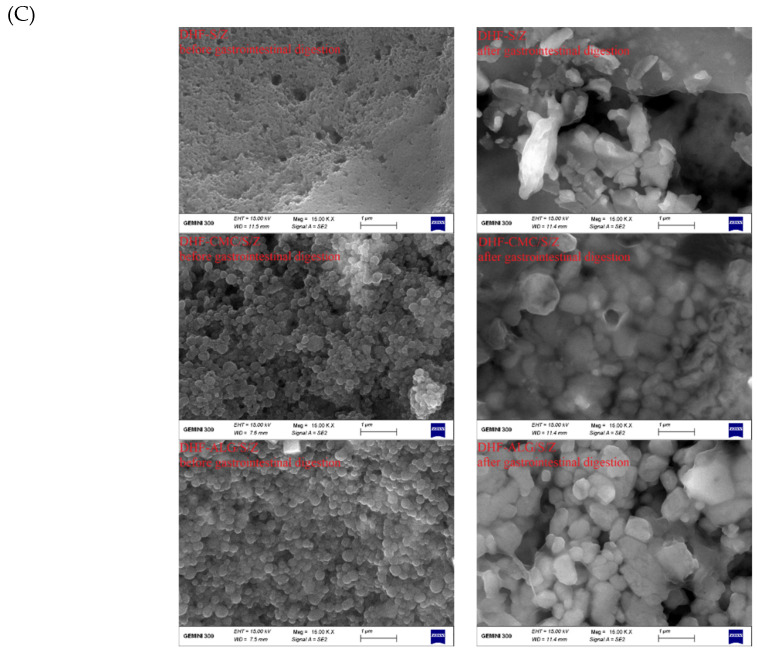
Influence of in vitro digestion time on the particle size (**A**) and bio-accessibility of DHF-S/Z, DHF-CMC/S/Z, and DHF-ALG/S/Z (**B**), FE-SEM images of DHF-S/Z, DHF-CMC/S/Z, and DHF-ALG/S/Z, 15,000× magnification times (**C**).

A gastrointestinal tract (GIT) model was applied to study the digestive fate and bioaccessibility of 7,8-DHF in different formulations. Particle size changes were monitored at a specific digestion time (30, 60, 120, and 180 min), and the results were presented in (Figure 9A). The mean particle size of DHF-S/Z was significantly increased after 60 min SGF digestion (*p* < 0.05). This finding was possibly due to the fact that the S/Z were exposed to ionic strength along with low pH and partially digestion via pepsase. The low pH and ionic strength exposure likely weakened electrostatic repulsion forces among the nanoparticles [52]. Particle size reduction in DHF-S/Z post-SIF-exposure was attributed to the fact that SIF contains bile salt with strong emulsifying ability. Bile salt can bind many biopolymer molecules and induce bridging flocculation [21]. The particle size of DHF-CMC/S/Z was increased after exposure to the stomach phase but remained relatively constant during incubation in SIF. This behavior suggested that the existence of CMC strengthened intestinal stability of DHF-CMC/S/Z. However, the particle size of DHF-ALG/S/Z remained fairly steady throughout simulated GIT, only showing a large increase at 30 min during the SGF incubation. The different influence of polysaccharide type (CMC or ALG) on gastrointestinal fate of colloidal carriers can be attributed to different molecular characteristics. FE-SEM microscopic observation further confirmed that exposure to simulated gastrointestinal conditions had a significant effect on the morphology of the 7,8-DHF-loaded nano-complexes (Figure 9C). For DHF-S/Z, irregular shapes have developed after they were added into the SGF and SIF, similar to an anomalous sheet structure. This shape acquisition was due to irregular aggregates appearing after SGF digestion. Furthermore, DHF-CMC/S/Z and DHF-ALG/S/Z exhibited a spherical shape. After gastrointestinal digestion, the nanoparticles possessed a relatively spherical morphology, resembling the cross-linked structure of large nanoparticles. Overall, CMC and ALG effectively protected the stability of DHF-S/Z nanoparticles through the GIT. Particularly, ALG performed extremely well.

After being exposed to simulated gastrointestinal conditions, 7,8-DHF bioaccessibility was measured after centrifugation and collection of micelle phases. As shown in Figure 9C, the S/Z was broken down when exposed to SGF digestion, and the core of 7,8-DHF was released, resulting in a low bioaccessibility. The bioaccessibility of DHF-S/Z gradually increased in SIF digestion, primarily due to the emulsibility of bile salts and sophorolipids. Another reason for this increase was the binding of hydrolyzed peptides of zein protein [33]. In the presence of CMC, low levels of bioaccessibility were exhibited in SGF digestion. This behavior was introduced by the vulnerability of CMC to acid, pepsin enzymes, and the alkali ions of SGF juices [53]. However, the bioaccessibility of DHF-CMC/S/Z was higher than that of DHF-S/Z, indicating a synergistic effect existed among CMC, sophorolipid and zein on controlling the release of 7,8-DHF. Furthermore, the presence of ALG further increased bioaccessibility to 75.46% (Figure 9B), showing the highest bioaccessibility of 7,8-DHF after GIT digestion compared to DHF-CMC/S/Z and DHF-S/Z. These results indicated that ALG increased the solubilization capacity of mixed micelles in small intestine fluids. Collectively, our results demonstrated that encapsulating 7,8-DHF in polysaccharide coated S/Z nanoparticles can promote an appreciable increase in its in vitro bioaccessibility.

## 4. Conclusions

In the present study, we compared the performance of ternary nanoparticles in the existence of two selected polysaccharides (CMC and ALG) for encapsulation of bioactive 7,8-DHF but with low bioavailability. CMC/S/Z exhibited lower PDI, particle size and turbidity, but higher zeta potential and loading capacity compared to ALG/S/Z. Furthermore, both polysaccharides supplementations promoted the EE value of 7,8-DHF in S/Z, especially CMC. Invitation of polysaccharides displayed positive effects on the formation and physical stability (pH and ionic strength stability) of ternary complexes. The formation of ternary complexes mainly occurred via hydrophobic effects, hydrogen bonding and electrostatic interactions. More significantly, compared to S/Z, ALG/S/Z, and CMC/S/Z obviously enhanced the storage stability and in vitro bioaccessibility of 7,8-DHF. CMC/S/Z possessed a higher storage stability for 7,8-DHF. In contrast, ALG/S/Z had a better in vitro bioaccessibility of 7,8-DHF. Collectively, the results of this study indicate that selected polysaccharides containing composite nanoparticles are efficient at encapsulating, retaining, and delivering 7,8-DHF, and might therefore be utilized in dietary supplements and functional foods. Future work will focus on the applicability of DHF-ALG/S/Z and DHF-CMC/S/Z in complex water-phase beverage systems. Besides, the transepithelial transport mechanism of DHF-ALG/S/Z in an Caco-2 cell model, and in vivo pharmacokinetic studies in rat will be also studies. 

## Figures and Tables

**Table 1:** EE, LC, particle size and PDI of 7,8-DHF in different colloidal systems.

Zein: 7,8-DHF (*w*/*w*)	Colloidal Systems	EE (%)	LC (%)	Particle Size (nm)	PDI
Without 7,8-DHF	S/Z	-		82.81 ± 0.61 ^a^	0.155 ± 0.010 ^a^
	CMC/S/Z	-		145.6 ± 0.75 ^c^	0.228 ± 0.006 ^b^
	ALG/S/Z	-		178.2 ± 0.35 ^d^	0.266 ± 0.024 ^c^
5:1	S/Z	82.42 ± 3.72 ^a^	7.49 ± 0.22 ^a^	114.7 ± 3.01 ^b^	0.271 ± 0.016 ^c^
	CMC/S/Z	88.63 ± 3.01 ^b^	7.38 ± 0.13 ^a^	177.4 ± 3.04 ^a^	0.363 ± 0.023 ^de^
	ALG/S/Z	84.15 ± 2.63 ^a^	7.01 ± 0.16 ^a^	214.3 ± 3.21 ^e^	0.394 ± 0.022 ^e^
10:1	S/Z	98.21 ± 1.31 ^c^	4.68 ± 0.40 ^b^	106.9 ± 1.11 ^b^	0.201 ± 0.021 ^b^
	CMC/S/Z	99.51 ± 0.24 ^c^	4.33 ± 0.12 ^b^	168.4 ± 3.62 ^dc^	0.334 ± 0.014 ^d^
	ALG/S/Z	98.71 ± 1.12 ^c^	4.29 ± 0.35 ^b^	200.1 ± 2.01 ^e^	0.352 ± 0.017 ^de^

Values are the means ± SD (*n* = 3). ^a–e^: Different letters in the same column indicate significant differences (*p* < 0.05) based on one-way ANOVA analysis followed by Tukey’s honest significant difference post hoc tests.

**Table 2:** Secondary structure of zein in DHF-S/Z, DHF-CMC/S/Z and DHF-ALG/S/Z.

Sample	Content (%)			
	α-Helix	β-Sheet	β-Turns	Unordered
DHF-S/Z	25.3 ± 0.23	25.8 ± 0.21	20.6 ± 0.19	28.3 ± 0.26
DHF-CMC/S/Z	24.7 ± 0.18	26.1 ± 0.17	20.1 ± 0.21	29.1 ± 0.20
DHF-ALG/S/Z	24.5 ± 0.16	26.6 ± 0.23	21.0 ± 0.26	27.9 ± 0.24

## Data Availability

Not applicable.

## Supplementary Materials

The following are available online at https://www.mdpi.com/article/10.3390/foods10112629/s1, Figure S1: The chemical structure of 7,8-DHF. Figure S2: Size distributions of nanoparticles with different zein to polysaccharide mass ratios, CMC/S/Z (A), ALG/S/Z (B). Figure S3: Size distributions of nanoparticles with different zein to polysaccharide mass ratios at pH = 4, CMC/S/Z (A), ALG/S/Z (B). Figure S4: The photograph of each colloidal particle at different pH conditions. Figure S5: The photograph of each colloidal particle at different NaCl concentrations.
