# Supplementary material for: Development, Characterization, Stability and Bioaccessibility Improvement of 7,8-Dihydroxyflavone Loaded Zein/Sophorolipid/Polysaccharide Ternary Nanoparticles: Comparison of Sodium Alginate and Sodium Carboxymethyl Cellulose
Source: Foods. 2021 Oct 29;10(11):2629. doi: 10.3390/foods10112629 (PMC8619035; doi:10.3390/foods10112629)
Supplement: Supplementary file 1 [file foods-10-02629-s001.zip › foods-1415499-supplementary.pdf]

Supplementary Materials

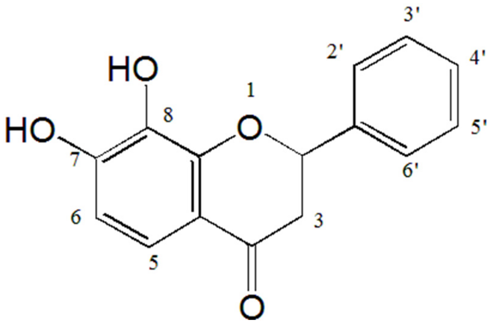

Figure S1. The chemical structure of 7,8-DHF.

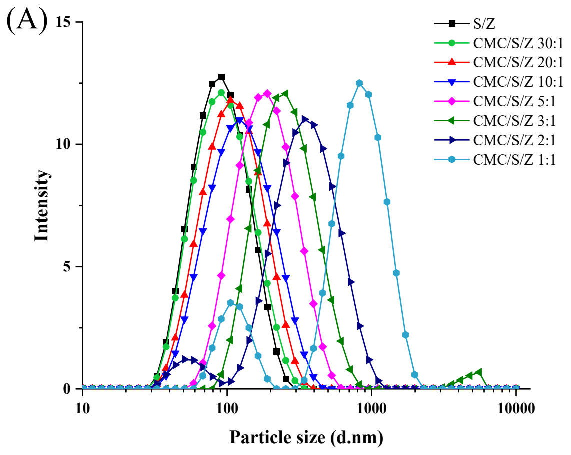

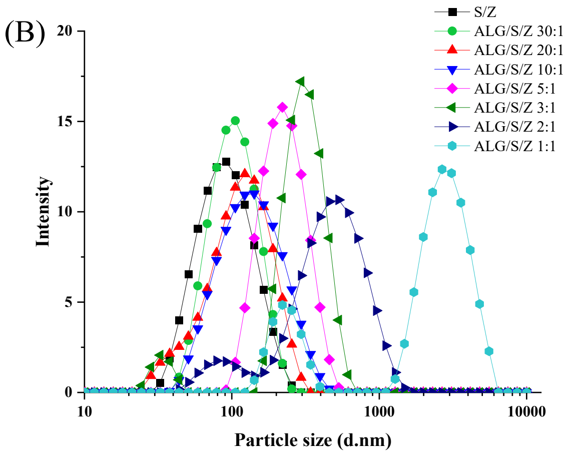

Figure S2. Size distributions of nanoparticles with different zein to polysaccharide mass ratios, CMC/S/Z (A), ALG/S/Z (B).

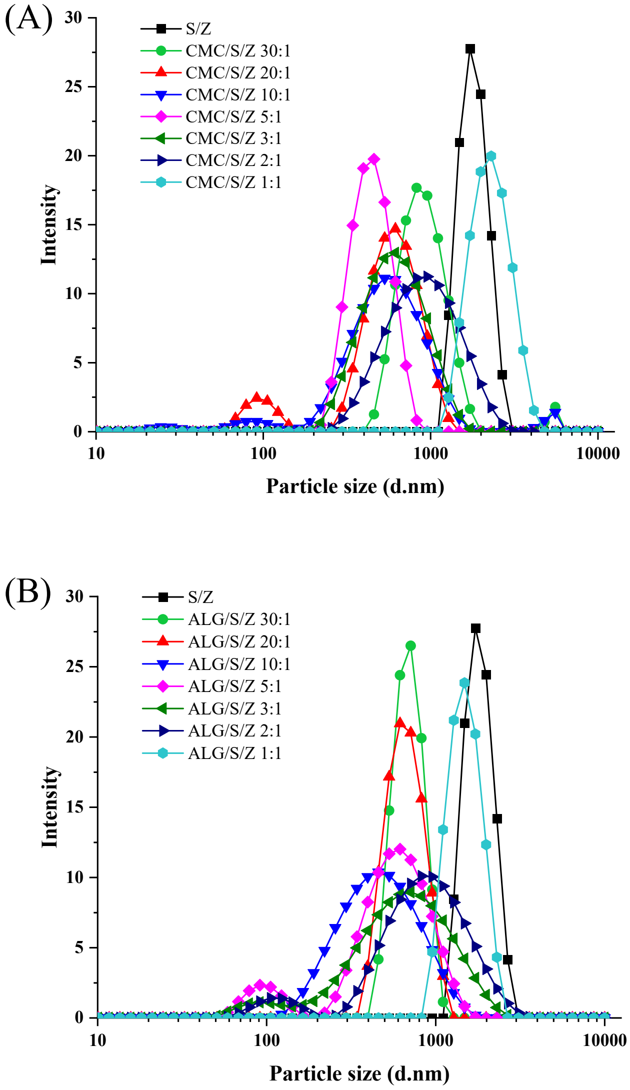

Figure S3. Size distributions of nanoparticles with different zein to polysaccharide mass ratios at pH=4, CMC/S/Z (A), ALG/S/Z (B).

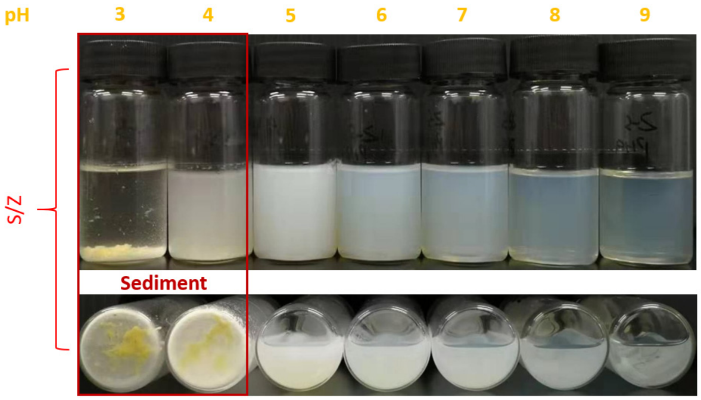

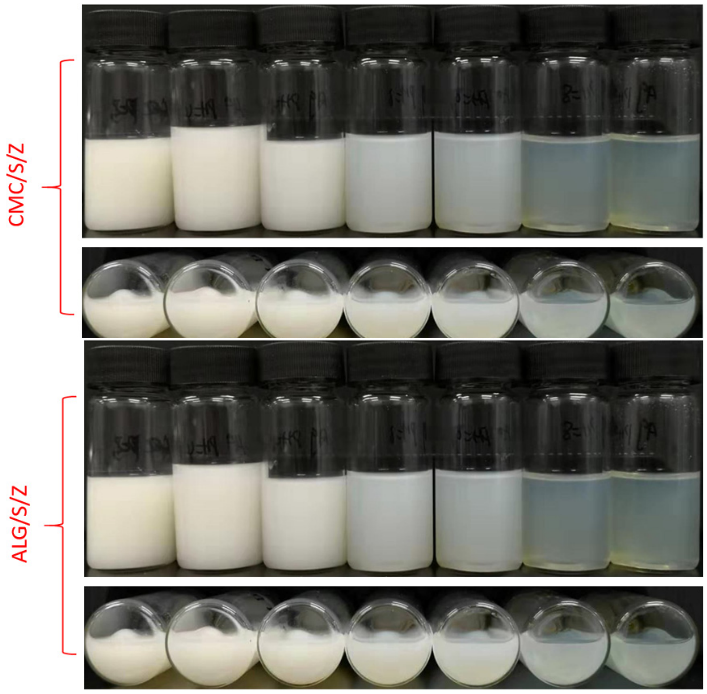

Figure S4. The photograph of each colloidal particle at different pH conditions.

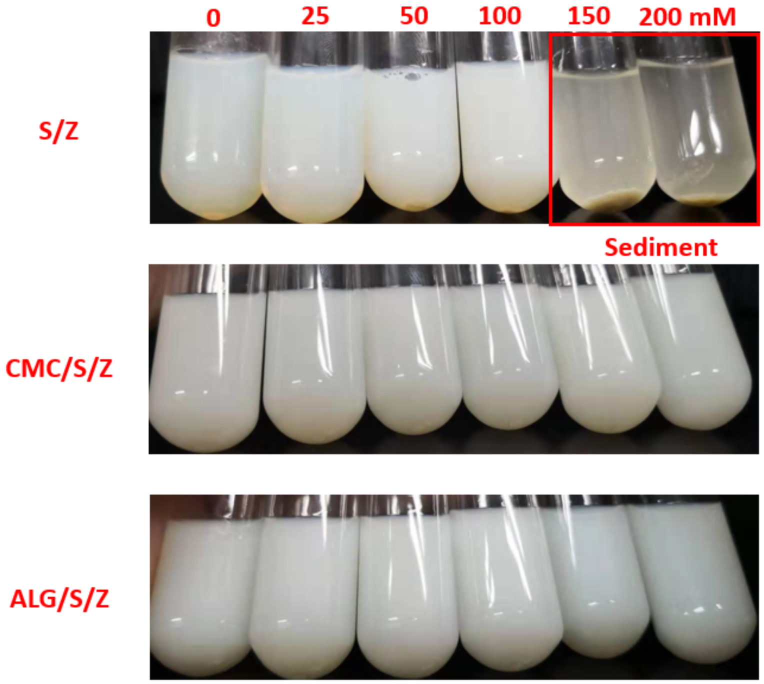

Figure S5. The photograph of each colloidal particle at different NaCl concentrations.
